# Clinical Report on Dysentery

**Published:** 1853-07

**Authors:** Austin Flint


### ART. III.— *Clinical Report on Dysentery. Based on an analysis of forty-nine recorded cases.* By Austin Flint, M. D.

> *"C'est en interrogeant fréquemment la nature que nous lui arrachons ses secrets."*

Among the notes that I have collected of diseases occurring in private and hospital practice, are embraced histories, more or less complete, of sixty cases of dysentery. On examination of these cases with a view to analysis, eleven were rejected in consequence of the patients having come under observation quite late in the progress of the disease, and the records being very imperfect. In two of these rejected cases the issue was fatal; in the others, either recovery took place, or the disease continued when the patient passed from under my observation. The residue of cases, after this elimination, amounted to forty-nine. These cases I have subjected to analysis. Taking up the several histories in succession, every thing noted in each history relating to the objects of inquiry contained in a series of sections, has been selected and arranged, each in the particular section to which it appropriately belongs. The sections are as follows:

1. Name. Age. Sex. Occupation. Season and year. Previous health and constitution. Duration of disease before coming under observation.
2. Circumstances attending the access of the disease.
3. Symptoms referable to the Digestive System.
4. Symptoms referable to the Circulatory System.
5. Symptoms referable to the Nervous System.
6. Symptoms referable to the Skin.
7. Symptoms referable to the Respiratory System.
8. Duration of disease to death or convalescence. Mode of dying. Relapses. Fatality.
9. Supposed causative agencies.
10. Subsequent health. Recurrence of the disease.
11. Treatment, and immediate (apparent) effect of remedies.

With the facts before me distributed after the foregoing sectional arrangement, I shall proceed to interrogate them in order to ascertain what deductions may be drawn therefrom bearing on the natural history, pathology, causation, and management of the disease. The object of this report is to present the results of such an investigation. What these results may be whether of much, or little interest and value, cannot be foreseen at this stage of the investigation, notwithstanding the familiarity with the details of the histories which is incident to the labor of transcribing them in making the preliminary analysis. Of this truth every one who has practiced similar analytical inquiries must be convinced. Were it at all necessary, the truth might be cited as conclusive evidence of the small value of even recorded, and still more of mere recollected experience, in contrast with the fruits of the study, by means of enumeration and comparison, of observations which have been accurately made, and carefully noted. The importance of what is distinguished as the numerical method of investigation has come, at length, to be very generally acknowledged. It is now admitted by most of those whose opinions are entitled to consideration, that, in the present state of medical science, we must mainly rely upon the results thus acquired as the basis of our knowledge of the laws of disease, of the events which make up their individuality, of their diagnostic characters, and, to a greater or less extent, of the effects of remedies. But the extent of the fallacies incident to reasoning from isolated cases, and to the general impressions drawn from what the memory retains of clinical experience, can best be appreciated by those who have tested the unreliability of their own opinions, thus formed, by submitting to the analytical process cases which they have themselves recorded. All who have had any practical acquaintance with this process, have doubtless been taken by surprise at some of the conclusions to which they have been led by it. And when we reflect upon the natural operation of the mind in the effort to generalize facts as they are irregularly and promiscuously presented in medical practice, trusting to the memory alone, the liability to error is by no means to be wondered at. To suppose that a practitioner of medicine will witness the phenomena of the diseases which he is required to treat, without indulging in speculative inquiries respecting them, is absurd. Such a supposition is hardly admissible. He will, and indeed must of necessity form, or adopt some theoretical notions of their character, causes, and of the best mode of managing them. He may honestly desire to try these notions faithfully by his experience, but it is plain he is not in the attitude of an unbiased seeker after the truth; and under the influence of his preconceptions the chances are many that his attention and memory will be occupied with facts which favor, to the exclusion of those which militate against these preconceptions. The results of this sort of experience are, in reality, apt to be altogether predetermined in the mind of the observer, and they prove just what it was expected they would, in consequence of this expectation. In view of the circumstances under which clinical observations are usually made, there can be no adequate security against hasty and illogical generalizations save in the accumulation of registered facts, and the analytical study of them; and when it is considered that not an inconsiderable share of past medical doctrines are only opinions based on the fallacious kind of experience just adverted to, it is not surprising that a host of traditionary errors are to be corrected by a more exact and logical method of investigation than has heretofore been pursued.

The limitations, and the liabilities to error in the prosecution of statistical researches, on the other hand, are not to be overlooked. The numerical method is not infallible, more than omnipotent. Its sphere of application is circumscribed. Its management affords scope for judgment and skill, and it may, therefore, be perverted. It does not consist wholly in arithmetical calculations. Its office is to supply data for ratiocination, and hence wrong inferences may be drawn from deductions which, abstractedly considered, are accurate. It is by no means designed to supersede other methods of study. It does not conflict with the labors of the microscopist, or of the chemical analyst, nor does it oppose, but only imposes a salutary check on efforts to discover the occult principles of disease, and to trace synthetically their connection with pathological phenomena. Indeed, it may be said to prepare the way for the latter, by narrowing the field of inquiry, and giving the proper direction to such investigations.

The introduction of the numerical method into the study of medicine is so recent, and the labor required in its prosecution is so great, that it would be far easier to enumerate diseases to which it has as yet hardly been applied, than to designate those concerning which all the information to be derived from this source has been exhausted. In the former category *dysentery* is to be included. Much as has been written on this disease, there are many questions pertaining to its symptomatology, etiology, pathology, and treatment, which can only be answered satisfactorily by means of statistical researches. The design of the writer of the present report, is to furnish a small contribution of facts and deductions bearing on some of these questions. The number of histories collected is not large, but they are sufficient to analyze in order at least that the results may be brought into comparison with those educed from other collections. With a view to such comparisons, as the writer has suggested and illustrated in another connection,* the plan of subjecting to analytical investigation, at different times, different series of cases, is preferable to waiting for a larger number, and embracing them all in a

* Clinical Reports on Continued Fever.

single analysis. Moreover, to procrastinate in labors of this kind is of course to incur the risk of their being never entered on—a risk enhanced by the increase of the task in proportion to the greater accumulation of cases.

It is proper to premise, what the critical reader will be at no loss to detect, that the histories in some particulars are uniformly defective, and that with respect to several points many are less complete than could be desired. In so far as positive or negative particulars are wanting, will, of course, the results of the analysis be limited. It is, however, one of the advantages of this method of investigating clinical observations, that, provided accuracy be observed in what is recorded, any deficiency in details in no degree vitiates the whole analysis. It is not less reliable on this account, carried as far as the data warrant. The only effect is to restrict the range of numerical research.

As already implied, in the *forty-nine* cases upon the analysis of which this report is based, are embraced only cases of acute dysentery, cases of the chronic form of the disease being excluded.

In eleven of the forty-nine cases the disease proved fatal. I may state here that the histories of all the fatal cases of dysentery treated by me in public or private practice, it is believed are embraced in this collection.* In every instance in which cases have passed under my observation without being recorded the patients have recovered.

### SECTION FIRST.

### *Age. Sex. Occupation. Season and year. Previous health and constitution. Duration of disease before coming under observation.*

*Age.* The ages, noted in forty-four cases, were between forty-eight and four years. Selecting several cases nearest each extreme, the ages respectively were as follows:

Of the ages nearest the maximum, 48, one case; 46, one case; 45, one case; 40, one case; 38, two cases; 35, four cases.

Of those nearest the minimum, 4, one case; 5, one case; 7, two cases; 8, one case; 10, one case; 11, one case; 18, one case; 19, four cases.

The mean age in the forty-four cases, is a fraction over 25 years.

It thus appears that, of the forty-four cases, in thirty the ages were between thirty-five and nineteen years. So far as these statistics go, they point to

* *i. e.* including the few rejected cases.

the period of life embraced between these years as that in which there exists the greatest liability to become attacked with dysentery, although other ages are not exempt.

The average age in ten of the eleven cases which proved fatal is a fraction over 27 years.

*Sex.* Of forty-three cases the number of males was twenty-four; of females, nineteen.

The fatal cases were distributed as equally as possible between the two sexes, viz., five males and six females. This, it will be noticed, gives a much larger fatality, proportionate to the number of cases, among the female, than the male patients.

*Occupation.* The occupations, in eighteen cases in which the records contain information on this point, were various. Females and children are of course excluded from this enumeration. Six were laborers, two were seamen; and of the following list, each occupation was represented by a single case: tinsmith, barber, tailor, farmer, currier, truckman, cigar maker, sail maker, overseer, editor, merchant.

*Season and year.* An enumeration of the cases which occurred in different months gives results which illustrate in a striking manner the decided predilection of the disease for a portion of the year. Of the forty-four cases, six were in July, seventeen were in August, nineteen were in September, and five in October, making the whole number of cases save one. The single remaining case was in March. None of the cases, thus, were in January, February, April, May, June, November and December. This law of the disease is of importance in its bearing on the subject of the causation of the disease.

As respects the prevalence of the disease in different years, the dates of the cases possess interest. Of the forty-nine cases, twelve occurred between 1838 and 1849; twenty-six in 1849, and the remaining eleven in 1850, 1851 and 1852. These results require some comment. It will be observed that up to 1849, for a period of eleven years, I collected only twelve histories. This number does not comprise all the cases treated by me during that period. It does, however, represent a fact which, in connection with these enumerations, is one of importance, viz., that during these years dysentery rarely occurred in this city. It did not prevail in any year as an epidemic, and sporadic cases were quite infrequent. This is stated as a matter of recollection; but inasmuch as during that whole period I was accustomed to make notes of cases occurring in practice, to a greater or less extent, I am certain that my records would have furnished a greater number of histories had the disease not been one of rare occurrence. It is proper to add, that, during this period, my opportunities for clinical observation in private practice were not small, and that for five years, in addition to private practice, I attended the County Almshouse, and the sick poor maintained at their homes at the public expense. The Buffalo Hospital of the Sisters of Charity was instituted in the summer of 1848, and in November of that year, sixty patients had been received. But a single case of dysentery was received from the time it was opened, to January, 1849. In the summer of 1849, when more than half of the cases embraced in this collection occurred, epidemic cholera prevailed to a great extent in this city. It appeared on the 30th of May, and ceased to prevail as an epidemic in the early part of the September following. The number of deaths from cholera during the three months intervening between the above dates, according to the reports made to the board of health, amounted to 2,501. In the month of September of that year, dysentery prevailed to a considerable extent. Fourteen of the twenty-six cases observed in 1849, were received at the Hospital of the Sisters of Charity in that, and the following months. The facts, as thus far considered, seem to authorize the presumption of some relationship between epidemic cholera and dysentery. Taking into view the remarkable immunity of this locality from the latter affection, even in its sporadic form, for a series of years prior to the year in which cholera prevailed, and its development in such close proximity to that epidemic, it would be reasonable to suspect that some elements involved in the genesis of these two diseases were common to both. The significance of statistical comparisons, indeed, might seem to be exemplified by such an inference. If, now, the attention be directed to the prevalence of dysentery in the years subsequent to 1849, it will be apparent that no essential connection is proved to exist between this disease and cholera. In 1850, 1851 and 1852, a small number of histories were collected. This is in a measure owing to the fact that after 1849 I ceased to attend at the hospital during the summer months, and I did not keep notes of all the cases occurring in private practice. On reference, however, to the hospital register during these years, I find that in the months of July, August, and September, of 1850, seventeen cases were received; in 1851, during the same months, six cases, and in 1852, for the same period, but a single case. Unfortunately for accuracy, in the hospital register for 1852, there are some omissions in the list of diseases, so that more than a single case may have been received; but that cases of dysentery were considerably less frequent in this year than in the two preceding years, is certain. This fact is all that concerns the present purpose, which is to compare the prevalence of this disease with cholera. In 1850 and 1851, cholera did not prevail to much extent, as an epidemic, in Buffalo. Cases occurred, now and then, in the course of the summer. Dysentery prevailed, but far less than in 1849. But in 1852 this city was again visited with a cholera epidemic attended by a fatality not less than in 1849. This season, however, was not again signalized by the prevalence of dysentery, cases of the latter disease being more infrequent than during the two preceding years when cholera prevailed but to a limited extent. It is thus seen, by extending statistical inquiries over the several years succeeding the season rendered memorable in this city by the occurrence of both cholera and dysentery in an epidemic form, that we are warranted in regarding the association of the two diseases only in the light of a coincidence. A survey of all the facts just presented, fails to establish any community in the causation of the two affections.

The liability to err in consequence of a limited range of statistical investigation is illustrated by the inference which accords with the results deduced from the analysis up to 1849 inclusive; and, on the other hand, the different conclusion arrived at by embracing the facts pertaining to subsequent years, affords an instance of the value of this method of determining truth.

In conclusion, it may be remarked, that in failing to discover in the facts before us any evidence of a connection between cholera and dysentery, so far as their origin is concerned, it is by no means implied that the latter affection may not receive important modifications from the association of the pathological conditions involved in both. This is a question which pertains to other branches of the investigation.

*Constitution and previous health.* Information on these points is contained in twenty-five histories. In twelve of these it is stated that the constitution and previous health were good. In thirteen cases it was otherwise. The facts pertaining to the subject in the latter cases respectively, and the issue of each case, are as follows:

No. 1. Labored under anæmia and spinal irritation incident to lactation, and had had hysterical convulsions two months previous. Recovered.

No. 2. A feeble woman, pallid, thin, had had delicate health for some time, and shortly afterward became affected with tuberculosis. Recovered.

No. 3. A dyspeptic. Recovered.

No. 4. A child four years of age. Had had several attacks of catarrhal croup, and during the previous summer had suffered from diarrhœa. Recovered.

No. 5. Been in delicate health for several years. A female lately married. Had hysteria shortly before the attack. Fatal.

No. 6. Subject to dyspeptic disorders. Recovered.

No. 7. Subject to hysteria. Recovered.

No. 8. A female aged about 28. Had presented evidences of scrofula. Recovered.

No. 9. Was recovering from subacute arthritis affecting the knee joint, which had confined him to the bed for several weeks, at the time he was attacked. Recovered.

No. 10. Had recently had epidemic cholera. Attacked eleven days after date of convalescence from that disease. Recovered.

No. 11. Had been in hospital four weeks before for intermittent fever and neuralgia, and was not fully recovered at the time of attack. Recovered.

No. 12. Had an attack of diarrhœa three months before, and in the mean time several recurrent attacks occurred. Recovered.

No. 13. Health delicate for one or two years before. Disordered menstruation and spinal irritation. Fatal.

From the foregoing facts it may be deduced, *first*, that, of a given number of cases, in at least one half, the constitution and previous health are good. *Second*, that there is no uniformity in character as respects the antecedent affections, or the disordered health. *Third*, that the constitution and previous health being impaired does not affect in a striking degree the prospect of recovery.

*Duration of disease before coming under observation.* The facts falling under this head are important only as showing to what extent the cases were under the personal observation of the reporter.

Of forty-three cases, in the histories of which the previous duration of the disease is noted, in twenty-seven the patients came under observation from the commencement, or so soon as the symptoms became sufficiently urgent to induce them to apply for medical aid. In sixteen cases the disease had existed for a greater or less period. The latter were, for the most part, hospital cases. Of these cases the disease had existed in two, two days; in four, three days; in one, four days; in two, five days; in one, six days; in four, seven days, and in two cases the disease had nearly advanced to convalescence, the patients being under treatment at the hospital at the time of commencing my term of service.

(To be Concluded.)

